# Psychosocial, emotional and professional challenges faced by female healthcare professionals during the COVID-19 outbreak in Lahore, Pakistan: a qualitative study

**DOI:** 10.1186/s12905-021-01344-y

**Published:** 2021-05-12

**Authors:** Sumbal Shahbaz, Muhammad Zeshan Ashraf, Rubeena Zakar, Florian Fischer

**Affiliations:** 1 Department of Public Health, Institute of Social and Cultural Studies, University of the Punjab, Lahore, Pakistan; 2 Department of Architecture, University College of Arts & Design, University of the Punjab, Lahore, Pakistan; 3 Institute of Public Health, Charité – Universitätsmedizin Berlin, Berlin, Germany; 4 Institute of Gerontological Health Services and Nursing Research, Ravensburg-Weingarten University of Applied Sciences, Weingarten, Germany

**Keywords:** Coronavirus, SARS-CoV-2, Women, Practitioner, Physician, Working environment

## Abstract

**Background:**

The novel coronavirus disease (COVID-19) is spreading rapidly, increasing the stress and challenges for healthcare professionals around the world. This study aims to discover the psychosocial, emotional and professional challenges faced by female healthcare professionals (HCPs) treating COVID-19 patients in Pakistan.

**Methods:**

Using an empirical phenomenological methodology, semi-structured telephone-based qualitative interviews were conducted with 22 female HCPs who were providing their expertise for COVID-19 patients in tertiary-level hospitals in Lahore, Pakistan. Purposive sampling was used for recruitment. The interviews were conducted between 20 July and 20 August 2020. The interviews were analysed using thematic analysis.

**Results:**

This study explored the psychosocial, emotional and professional challenges faced by female HCPs serving COVID-19 patients. Five themes were observed in the interviews: apprehension while treating COVID-19 patients; feelings towards COVID-19 patients; challenges as female HCPs and coping strategies; confidence in government, administration and self-reflection; and finally, future concerns and recommendations. Many of these themes have also been linked with cultural issues, making the results specific to Pakistan.

**Conclusions:**

During the COVID-19 pandemic, female frontline HCPs have faced immense psychosocial pressure, ranging from unsupportive family norms to an unwelcoming working environment and insensitive hospital administrations. Moreover, rumours among the general public, lack of proper training, missing incentives and improper system surveillance have increased the anxiety and stress among HCPs. Hence, legislators are advised to take appropriate actions countrywide in order to alleviate the still ongoing challenges and support female HCPs in their working environment.

**Supplementary Information:**

The online version contains supplementary material available at 10.1186/s12905-021-01344-y.

## Background

The novel coronavirus (COVID-19) has spread very quickly around the globe. The pandemic has led to many acute cases of infection, but is also associated with adverse long-term health consequences. In several countries, including Pakistan, it has almost led to a collapse of the healthcare system [1, 2]. This highly contagious virus imposes an enormous scale of infection, and exerts massive pressure on governments, medical institutions and healthcare professionals (HCPs) [3, 4]. Like other coronaviruses, i.e. SARS and MERS, inter-human transmission of COVID-19 typically occurs via aerosols [5]. Thus, the COVID-19 pandemic is undoubtedly the largest outbreak of atypical pneumonia since the SARS outbreak of 2003, with the number of cases unrelentingly escalating beyond geographical restrictions [6].

Pakistan is surrounded by highly affected countries like China, which was the first one to experience this outbreak, and Iran [7]. According to data from Johns Hopkins University, the highest numbers of incident cases and deaths due to COVID-19 in Pakistan were observed in June 2020 [8], the month before this study was conducted. Within the province of Punjab, the city of Lahore has been the epicentre, with the highest number of confirmed cases. Therefore, the government initially introduced social distancing and ordered lockdowns (e.g. the closing of all educational institutions) in March 2020 [9]. The lack of standard operating procedures and poor healthcare infrastructure for the management of a pandemic put a lot of strain, in the form of increased numbers of COVID-19-positive cases, on an already weak healthcare system in Pakistan [10]. Subsequently, the government established local guidelines for combating COVID-19 in collaboration with the Corona Experts Advisory Group (CEAG) aimed at improving the healthcare system and protecting HCPs in public hospitals. These measures included training sessions for healthcare professionals for managing the pandemic, the proper handling of personal protective equipment (PPE), the isolation of HCPs for two weeks after working for one week in high-risk places (COVID-19 wards and intensive care units), and allowing only necessary personnel on alternate days in other specialities to minimise exposure [11]. Nevertheless, the government not only failed to provide these facilities but took drastic measures to stop HCPs from striking, instead of understanding their concerns [12].

This situation was associated with increased psychological stress among the general population, and among HCPs in particular [13]. Due to their close contact with COVID-19 patients, healthcare professionals were at greater risk of developing psychological and stress-related disorders than the general public. The reason for this psychological stress could be excessive workload, deficient PPE, negative broadcasts about HCPs’ work ethic and updates regarding COVID-19 [14]. In addition, along with increasing social and emotional pressures, moral quandaries, rapidly evolving unfamiliar practice-setting and inadequate provision of facilities for mental catharsis could augment the risk of increased stress level among HCPs [15]. These could also be referred to as psychosocial risk factors at work, which greatly affect professional capacity in addition to the social, physical and mental wellbeing of HCPs [16, 17].

To reinforce emergency preparedness at the national and community levels, the government of Pakistan issued orders to shut down outpatient treatment as well as elective surgical facilities [18]. Qualified HCPs are essential in such a situation. However, to ensure the efficient training of the healthcare workforce during the pandemic, not only are ample numbers of HCPs required, but they also need enhanced abilities to deal with the higher inflow of patients [19]. For all of this, their wellbeing and safety are vital if they are to render unrelenting and advanced patient care, in addition to combating the outbreak [20].

In order to develop an effective support system for HCPs, it is critical to understand the core sources of anxiety and to address them, rather than opting for generic approaches to stress reduction. These sources of anxiety may be different cross various settings, but they can erode the confidence levels of HCPs, not only in themselves but also in the healthcare delivery system, which could worsen this grave situation. Targeted approaches need to be developed to address these apprehensions and deliver context-specific support to HCPs [19].

In Pakistan, women are an indispensable part of the healthcare system as more than 50% of all HCPs are women, serving as doctors, nurses and allied health professionals in all corona-dedicated hospitals within the country [21]. On the one hand, female HCPs have sacrificed their own needs to contribute to combating the pandemic and have shown the highest levels of professional commitment [22]. On the other hand, they have faced emotional and psychological stress as well as social isolation and abandonment because of the health threats [23]. Indeed, the outbreak of COVID-19 in Pakistan carries grim challenges for all HCPs, but female HCPs experience more problems because of their dual responsibilities, that is, at home as well as in the workplace. There is a paucity of literature regarding the psychosocial, emotional and/or professional challenges faced specifically by female HCPs in Pakistan, particularly during the COVID-19 pandemic. This qualitative study aims to describe the experiences and challenges faced by female physicians, nurses and allied health professionals caring for COVID-19 patients during the outbreak.

## Methods

### Study design and participants

The qualitative methodology [24] was opted for analysing the psychosocial, emotional and professional challenges faced by female HCPs treating COVID-19 patients in Pakistan. This scientific approach emphasizes the feelings and practices of participants and provides a subjective and insider perspective on the challenges they had to face [25]. We focused on HCPs who had only received basic infectious disease management training during their medical education, but had no practical experience of infectious disease management before the pandemic. The study participants included those who had directly treated COVID-19 patients in Lahore, i.e. in the Services Hospital and Mayo Hospital. Using purposive sampling, 22 female HCPs were selected, including doctors, nurses and allied health professionals. They were asked about psychosocial factors, including social support, feelings of loneliness and helplessness, stresses in the work environment, the organizational culture of the hospitals where female HCPs work, civility and respect for HCPs, recognition, influence and social disruption, in addition to emotional and professional challenges. Participants were recruited until the data saturation point was achieved.

### Data collection and analysis

Data was collected through in-depth interviews (IDI) conducted by telephone with study participants [26] in order to maintain social distancing. Prior to the interview, a time was fixed for the call and the study objectives were explained to participants. The anonymity and confidentiality of the study participants were ensured. Verbal informed consent was taken before the start of each interview. Voice recordings were made with the respondents’ permission.

We used a semi-structured interview guide for data collection (Additional file 1), which was developed for this study. The guide started with general questions related to the participant’s age, marital status, number of children, and the duration of duty on COVID-19 wards. Afterwards, comprehensive open-ended questions were used to access insider perspectives on questions like: “How does taking care of COVID-19 patients differ from caring for routine patients?”, “What are your core psychological concerns about treating COVID-19 patients?”, “What kind of training did you receive before treating COVID-19 patients?”, and “What challenges did you encounter and what were your coping methods?”. To gain a clearer picture of the challenges related to psychosocial aspects, we used the following questions: “How did you manage your work and family life during the pandemic?”, “In what ways did you receive support from your family?”, “What societal pressures have you faced?”, “Have you or any of your family members been infected?”, “Who helped you during repeated quarantine periods?”, and “Did you receive any stress-releasing therapy or assessment?” Probing questions (such as “Please elaborate…” or “Please explain…”) were also used. Each interview lasted about 30 to 40 min and was transcribed immediately after its completion.

The analysis was undertaken according to qualitative techniques. The transcripts were read several times to identify themes and sub-themes. At that point, all the material was reviewed and summarized according to the outlined themes by two coders. A thematic analysis was conducted in order to draw conclusions [27].

Ethical approval was obtained from the ethical review board of the Department of Public Health, University of the Punjab, Lahore.

## Results

The study sample comprised 22 female HCPs. All the married participants (n = 17) were living with their families, and of the remaining five participants who were unmarried, three lived in a nursing/doctor’s hostel. A summary of the socio-demographic characteristics of study participants is provided in Table 1.

**Table 1:** Socio-demographic characteristics of study participants (n = 22)

**Characteristics**	**n**	**%**
Type of HCPs		
Doctors	9	40.9
Nurses	8	36.3
AHPs	5	22.7
Age		
25–30 years	8	36.3
31-–35 years	12	54.5
> 40 years	2	9.1
Marital status		
Married	17	77.2
Single	5	22.7
Number of children		
0	3	17.6
1–2	9	52.9
3–4	5	29.4
Years of work experience		
3–5	8	36.3
> 5	14	63.6

This study explored the psychosocial and emotional challenges and lived experiences of HCPs caring for COVID-19 patients. Their interviews outlined the following five themes that were common to the entire group of study participants: apprehension while treating COVID-19 patients; feelings towards COVID-19 patients; challenges as female HCPs and coping strategies; confidence in government, hospital administration and self-reflection; and finally, future concerns and recommendations. Many of these themes have also been linked with cultural issues, making the results specific to Pakistan.

### Apprehension while treating COVID-19 patients

All of the study participants were concerned about the contagiousness of the virus, not just for themselves but also for the people around them. The aggressive behaviour of the virus, causing unanticipated deaths of even very young physicians, made them feel that they were immersed in a crisis and would not be able to cope with it. Most of the married participants were very concerned about their children and parents, because treating COVID-19 patients could have made them carriers of the virus. This situation was crucial for HCPs who were parents because they had to return home daily where their children were present and waiting for them. Another fear for some of the study participants (n = 7) was post-duty quarantine. This is exemplified in a statement made by one participant:

Working with COVID-19 patients in intensive care units was more like working in a morgue. You can feel death everywhere. Somehow you manage to work during duty hours, but when you’re alone it’s hard to collect your broken pieces. You have no shoulder to cry on and no one to boost you.

This feeling of loneliness and helplessness made quarantine even harder than the duty itself. Some respondents claimed they were having difficulties in arranging help to provide them with necessities (related to food, supplies etc.) during post-duty quarantine, while others complained about the lack of emotional and social support for female HCPs. One of them added:

As I quarantined myself in an apartment, the biggest problem I faced was lack of support. If I needed some groceries or supplies nobody was ready to come and provide me with that, I had to go out and get things myself. This always made me worried: what if I’m a source of spreading the virus?

Those whose partners had a non-medical profession retreated from them (during their COVID-19 duties), not only physically but also emotionally. The respondents said that their partners had the feeling that they prioritized their jobs over their own family’s health and safety. Some respondents who did not have children preferred to stay away from home during their COVID-19 duties, because they were afraid of getting blamed for carrying the disease to their in-laws’ homes. Those who had partners in the same professions reported receiving more emotional support. However, they failed to manage post duty quarantine during or after their COVID-19 duties besides getting leaves for isolation, as they were living with their families all the time, burdened with additional household chores and nil support from their spouse. This situation was elaborated in the following way:

We’re both doctors and can’t leave our kids with some third person, so I had to stay with my kids while my husband quarantined himself after duty

Another respondent added:

The free days for post-duty quarantine were even more difficult. Firstly you have to stay with your kids as nobody was ready to take care of them, secondly for the safety of the family we let all the maids or helping hands go, so I had to do all the household chores which were previously done by maids.

Only unmarried participants received full support from their families and maintained the recommended quarantine during and after their duty period. All the study participants acknowledged that their parents and siblings were a constant source of support for them.

Initially, social support was provided by the general public, but this deteriorated over time due to fake rumours about COVID-19 deaths. For example, it is rumoured that physicians are killing COVID-19 patients by giving them poison or selling their organs. One participant confessed that people even asked her about how much money she gets for every death. One other claimed:

People got aggressive during the later stages when we advised them to go for COVID-19 testing and assumed that we were planning to kill their patients. As a woman, it’s really difficult to counsel people when they start to misbehave*.*

Some participants claimed that people with educated relatives or people around them were appreciated for their efforts while combating the pandemic. In contrast, others received negative outpourings all the time. All the participants concluded that their social circumstances were better initially, when people were afraid of this pandemic. However, later on, although the number of cases and deaths increased, the fear, care and social distancing decreased due to negative rumours and circulating tales about COVID-19 deaths. This has been reinforced by social media, which not only increased work pressure but also stress and health concerns.

### Feelings towards COVID-19 patients

All the HCPs felt depressed while treating COVID-19 patients due to the frequently unexpected response of the virus and treatment guidelines that were different from usual, making it a professional challenge. One of the study participants reported:

We were told not to resuscitate and not to ventilate patients in the intensive care unit. That was quite frustrating for me as I always got a feeling that it’s an injustice to the patient.

Another participant said:

I once resuscitated a young patient in the intensive care unit, and he gave a positive response. I was so happy, but my colleagues gave quite a backlash instead of appreciating me, asking why I would endanger myself for this patient and that I’m setting a bad trend.

Another respondent explained:

Despite my experience, I had no idea how this virus would work with anybody. One moment, a patient’s FiO_2_ [Fraction of inspired oxygen] was improving, the next moment he was gone, which was really depressing – being a competent doctor.

Another study participant said that counselling attendants (family members of patients) was the most difficult task for female HCPs, because attendants were not allowed to see their relatives. The speed with which the disease progresses in some people made their attendants feel that the patient had been fine and the hospital staff killed him, which made them behave badly towards the healthcare personnel. This is exemplified by the following statement.

If you were treating a patient for the last 12 hours who is getting better during your shift, and two hours after the shift change you learn that he died, you get a feeling that he was being mistreated. Maybe you could have changed this if you had stayed longer. In reality, things would have remained the same, but you keep on blaming yourself and others.

On the other hand, those who were on duty at the time of death were afraid of being bullied by the patient’s family. So in either case, it is traumatic. Some HCPs claimed that the lower rate of survival among the intensive care unit’s patients was due to the carelessness of senior consultants. One study participant said:

We were only supposed to follow instructions given during rounds, but were not allowed to make decisions for patients. In case of emergency, we had to inform seniors with duty on call and by the time the senior arrived, the patient had died.

Some of the HCPs claimed that a lack of psychological support made patients more anxious, and they developed shortness of breath due to both the overwhelming depression caused by COVID-19 and the loneliness. In contrast, others referred to a lack of air conditioning on COVID-19 wards and in intensive care units, along with hot and humid weather as a reason for the shortness of breath.

A few study participants claimed that power inequalities reached a peak during the coronavirus pandemic:

The relatives of powerful people got beds in intensive care units, even if they didn’t need it, or had their beds booked for emergency situations, whereas deserving ones suffered.

One participant also complained about the lack of portable equipment like dialysis machines and echocardiography for COVID-19 patients, which increased the preventable death toll. All participants had multiple concerns about treating COVID-19 patients, which reduced their stamina and courage to work through these difficult times.

### Challenges as female HCPs and coping strategies

Almost all the study participants faced additional challenges related to being a female HCP. The majority had faced major transportation challenges due to lockdowns and the unavailability of either public or private transport. For that reason, they experienced additional family arguments due to requesting their spouses to drop them off at work every day, because the majority of the women neither owned a car nor knew how to drive. All the participants with children faced additional challenges of maintaining family chores along with COVID-19 duties. For example, housemaids were not permitted to work at their homes owing to fears of the virus being transmitted through them, because most maids work in multiple houses. Moreover, home-schooling and online classes for children without any help increased the conflicts related to work-family balance during their COVID-19 duties. One participant explained her concerns in the following way:

My child is just eight months old and it was impossible for me to stay away from him as I had nobody to look after him.

Another one claimed:

Nobody in my family was ready to take responsibility for my three kids. So, I had to be with them.

The challenge of families becoming infected was highlighted by another HCP:

My whole family was COVID-19 positive due to my repeated duties with COVID-19 patients and staying with them without being quarantined.

Most of the married participants said that their spouse refused to take family responsibility alone. However, the situation was also tough for unmarried staff residing in hostels, because during their post-duty quarantine period, or if they tested positive for COVID-19, they were ordered to leave the hospitals hostels. The study participants pointed out, that male HCPs could stay in hotels or private facilities for quarantine, but female HCPs cannot do so as it is culturally unacceptable and considered unsafe for females to stay alone at a hotel or new place. Instead of being shown gratitude and support during these difficult times, they were thrown out of their hostels by the administration; and by moving to their residence in other cities they became carriers to others as well.

Another problem faced by the participants was a lack of changing rooms for women. Following cultural norms, 14 participants wore the *hijab* or *dupatta*, which are scarves that some Muslim women wear to cover their hair and neck. But after removing their PPE, they had to go without wearing this kind of scarf, which was quite uncomfortable for them. In one facility, not even a washroom was available where they could go before leaving the hospital. All the married respondents said that they had the extra burden of home-schooling their children in addition to basic household chores that were previously done by maids.

Another serious challenge highlighted by female HCPs was inadequate security in hospitals. Especially those who worked the night shift were badly treated by the attendants of deceased patients, and hospital security failed to ensure their safety. On participant explained:

I hid the whole night in a nearby ward as the attendants of patients were beating all the staff when we broke the news of their patient’s death.

Another one said:

Due to the negativity spread among the general public during the later stages, one night a few attendants broke down the door of the intensive care unit and we ran to save our lives. Hospital security failed to give protection, so finally we called the police, who saved us.

These circumstances increased the fear and anxiety among HCPs, but not a single one of the study participants tried any psychological counselling or assessment as a stress-relieving strategy. All the participants increased their praying and tried to make their bond with Allah strong as a form of meditation. The majority of female HCPs also felt better after crying a lot alone when they were tired of battling with this situation. Moreover, the health authorities also paid no attention to the mental health of HCPs, and there were no workshops or sessions about psychological coping with the ongoing pandemic crisis. This was judged as an ignorant and irresponsible act by the interviewees.

### Confidence in government, hospital administration and self-reflection

As a vital part of the female HCP team, the nurses faced the extreme issue of pregnancies among other HCPs during the pandemic, which was spoken about by a few study participants:

Despite government policies of not putting pregnant ladies and staff with comorbidities on COVID-19 duties, hospital administration forced them to do duties and threatened them with termination if they refused.

Despite being pregnant I was forced to undertake duty in COVID-19 intensive care units. And when I tested positive for COVID-19, the administration refused to give me quarantine as I had mild symptoms. I kept on working all the time while I was COVID-19 positive.

One participant claimed that her miscarriage was a result of the hospital overburdening her with COVID-19 duties. All participants revealed their cynicism about health departments due to a lack of training facilities for dealing with COVID-19 patients. The only training held by the authorities was during March 2020, in the form of a session involving a few senior specialists from each department. One respondent explained:

The training session was very general. It was not COVID-19 oriented or COVID-19 specific at all.

Another participant further elaborated:

The training included those seniors who never bothered to care for COVID-19 patients or explain it to other members of the department.

One HCP who took an up-to-date training session while working in a reputable private hospital during the early days of the COVID-19 pandemic said:

None of the staff at the government facility, including medical and paramedical staff, had any idea about the right way of donning and doffing personal protective equipment, which led to increased corona-positive cases among them.

Most of the participants had a positive attitude towards the efforts the government made to combat the pandemic. However, all of them were very disappointed with the hospital administration, which failed to provide good-quality treatment facilities. There has been a lack of facilities for HCPs who become COVID-19 positive while treating patients. This is illustrated by the following statements:

There wasn’t even any separate testing area for HCPs. They had to use links to get a bed in hospital if they got sick.

If an HCP tests positive while working on a COVID-19 ward, he gets 15 days quarantine period. But if he tests positive after it, the administration refuses to give rest, deducts pay even after providing COVID-19 reports and gives them extra duties after they return to work.

All of the respondents claimed that they received no additional health allowance, even after several announcements from the government:

My colleague became COVID-19 positive while caring for patients. Her family opted for a private facility when she needed intensive care, but the government offered no health allowance.

Due to public announcements of salary incentives by the government, the general public have the impression that doctors are getting a lot of reward for their services, whereas in reality not a single one of us got a single rupee incentive.

All the study participants demanded checks and balances from the government about on-the-ground realities of what was going on in hospitals. One respondent explained:

Seniors and influential people in the departments just came, took photographs and took the credit, while those juniors who actually did the work did not get any gratitude or acceptance from the administration. This increased their depression and decreased their motivation to work.

Another added:

There must be checks and balances at every level so that those who have worked get credit for their hard work, and those who didn’t become motivated to work next time. But the situation is the opposite of that here.

Most of the study participants claimed that this epidemic had increased their confidence in themselves and their competence. A few claimed:

I never imagined that we would be facing such a pandemic in this era or that I would be the one saving others’ lives. This has increased my passion for this noble profession.

*This pandemic gave us a chance to polish our skills and expertise.*

*Although it’s a challenging time, it made us prove ourselves, not just in front of others but to ourselves.*

Most of the participants explained that, even though the pandemic prevention duty was hard, it triggered a process of introspection and self-analysis. Therefore, it strengthened their determination, revealed their true potential, and augmented their motivational spirits.

### Future concerns and recommendations

Almost all of the study participants claimed that the pandemic made them realise the importance of living “today”. This is illustrated by the following statements:

We’re never grateful for what we have and keep on planning for a better future. This pandemic has made us realise the importance of health, family and self-care.

This pandemic made our belief strong in the supremacy of God almighty as a lot of professionals had their plans to emigrate, to go for vacations. But they had to surrender in the face of God’s will.

All respondents agreed that there must be a disaster plan in case there is a second wave of COVID-19. The participants emphasized:

The response should be fast, special recruitment for coronavirus came after the peak months of the epidemic, due to which they suffered an additional load.

Protective gear was rare during the initial days. And fear was at its peak, which created a lot of pressure on healthcare professionals. Therefore, the government should keep it [PPE] now for any emergency situation in the future.

Almost all of the study participants suggested that the government should reward those who have worked during the pandemic. This relates not only to financial issues, but also as a form of appreciation, which would increase their courage and dedication towards work.

Moreover, they stated that improving the ratio between patients and HCPs and appropriate guidelines and recommendations to handle epidemics must become a regular part of medical training. One participant highlighted this issue in the following manner:

The drill to work as a team should be improved. We know how to work as a team in our respective departments, but working collectively as a team with different departments must be learned and improved.

Despite tough circumstances and challenges, 13 of the 22 respondents felt happy to have served during the COVID-19 pandemic. Firstly, they had the feeling that they had fulfilled the oath they took to serve mankind. Secondly, their self-confidence had received a huge boost due to working in such difficult times.

## Discussion

This study highlights the psychosocial, emotional and professional challenges faced by female HCPs during the COVID-19 pandemic in Pakistan. HCPs are always at the forefront, putting their lives at risk for the health of the community. Our study found that caring for patients with COVID-19 created mental and social discomfort for female HCPs, which is in accordance with other international studies [22, 23]. In other countries, intense working conditions, the enormous patient load, and shortages of protective gear were the main source of anxiety among HCPs [6, 23]. Our study highlights that apprehension about family was the main challenge experienced by female HCPs in Pakistan. Furthermore, difficulties created by the hospital administration led to further challenges, resulting in higher stress levels, whereas in other studies the hospital administration proved to be helpful, specifically for female caregivers [23].

Physical fatigue, psychological vulnerability, (perceived) health risks to their families, and a lack of adequate training, equipment and facilities, in addition to lack of support from society, led to undesirable emotions, such as anxiety, distress and helplessness, which have already been described in previous studies [28, 29]. This study shows that HCPs – no matter whether they are physicians, nurses or allied professionals – experienced significantly higher negative sentiments about their personal health directly after the pandemic started. Therefore, timely psychological interventions are needed for female HCPs, as in other countries, where meditation and relaxation therapies have been used to release increased stress [23, 30]. Moreover, it is important to provide facilities both during and after duty quarantine for female HCPs so they can avoid carrying the disease to their families and communities [31, 32]. Our study results reveal that, after working with COVID-19 patients and not being quarantined due to family and societal responsibilities, female HCPs were exposed to extreme frustration, fear of transmitting the virus, and clashes between their commitments to family and their occupation. In addition, due to no safe quarantining facilities or health insurance/incentives and safe working environments being provided for female employees, feelings of resentment and disappointment were seen among all the female HCPs [33].

This study highlights the immediate need to start appropriate training sessions in how to combat pandemics for all HCPs, and specifically for nursing staff because they are responsible for advanced patient care and form a strong link between consultants and patients at every level [33]. Furthermore, our results emphasize the need to improve health policies in order to provide immediate facilities as well as checks and balances to promote and acknowledge those who have worked hard, instead of those who have personal links with the hospital administration. Moreover, the health of comorbid and pregnant HCPs should be given serious attention, to decrease their risk of infection. At the same time, adequate infant and childcare facilities should be provided for the families of female HCPs so that they can become a more effective part of the healthcare system.

However, due to the existing circumstances, the participants felt distressed and accused themselves of infecting their families [34]. The insider perspective provided by these HCPs reveals the need for direct communication between the government and care-providing HCPs. The current study also indicates a constant need to inform people about the actual situation during a pandemic and to combat local rumours that create a negative impression of already suffering and struggling HCPs [35].

In addition, numerous studies have revealed that outbreaks are a cause of psychological distress for female care providers [19, 36]. Therefore, a proper system of psychological evaluation must be developed in Pakistan. Positive coping styles, in combination with social support, must be mediated for releasing stress. Self-reflection techniques need to be promoted to boost professional identity [23, 37]. Moreover, female HCPs need a place and time for promoting psychological growth and positive psychological adjustment [23, 33] so that they can be a better part of the healthcare and family system.

Various studies have revealed that proper health facilities and health allowances for HCPs would improve their level of trust in the authorities, as well as their willingness to work during such difficult times [37, 38]. The same goes for female HCPs in Pakistan, a proportion of whom (nurses, technologists) never receive any of this. The testing and treatment of female HCPs, along with their family members, should be offered without discrimination, as in other countries [39]. Hence, the safety of female HCPs should be prioritized, by means of up-to-date training, health education, adequate duty and quarantine periods, health incentives and insurance, the availability of sufficient protective gear, and psychological provision [40, 41].

## Limitations

The limitations of this study are mainly related to the purposive sampling. This study does not aim to be representative, but it has provided valuable insights into the experiences of female HCPs. Due to the extremely contagious nature of the coronavirus, and in order to prevent cross-infection, we were not able to conduct focus group discussions, which would have opened up further valuable avenues to explore. Several of our results are closely associated with cultural factors. For that reason, this study is specific to the Pakistani context. However, it would be advisable for future research to include these cultural aspects in order to understand the challenges and responses towards COVID-19 in a better way.

## Conclusions

This research has provided an insider perspective on the psychosocial, emotional and professional challenges faced by female HCPs in Pakistan during COVID-19. The results demonstrate that female HCPs are psychosocially challenged by society and the hospital authorities. The unavailability of support for their children put not only female HCPs at risk, but also their families and the wider community. Due to inadequate post-duty quarantine techniques and facilities, in addition to transport problems and dependence on their spouses for commuting, their mental health is seriously compromised. Psychological and financial support, in addition to health insurance, should be offered to female HCPs by governmental and non-governmental institutions. Therefore, health policy reforms are needed in order to be prepared for possible further health emergencies in the future in order to maintain the stability of HCPs’ families, as well as their willingness and ability to work.

## Supplementary Information

**Additional file 1**. Interview guide.

## Data Availability

Data is available from corresponding author upon reasonable request.

## Abbreviations

CEAG: Corona experts advisory group
COVID-19: Coronavirus disease 2019
HCP: Health care professional
PPE: Personal protective equipment

## References

[1] Sahin AR, Erdogan A, Agaoglu PM, Dineri Y, Cakirci AY, Senel ME, Okyay RA, Tasdogan AM (2020). 2019 novel coronavirus (COVID-19) outbreak: a review of the current literature. EJMO.

[2] Waris A, Khan A, Ali M, Ali A, Baset A (2020). COVID-19 outbreak: current scenario of Pakistan. New Microbes New Infect..

[3] Wang C, Pan R, Wan X, Tan Y, Xu L, Ho CS, Ho RC (2020). Immediate psychological responses and associated factors during the initial stage of the 2019 coronavirus disease (COVID-19) epidemic among the general population in China. Int J Environ Res Public Health.

[4] Wang C, Pan R, Wan X, Tan Y, Xu L, McIntyre RS, Choo FN, Tran B, Ho R, Sharma VK, Ho C (2020). A longitudinal study on the mental health of general population during the COVID-19 epidemic in China. Brain Behav Immun.

[5] Chirico F, Sacco A, Bragazzi NL, Magnavita N (2020). Can air-conditioning systems contribute to the spread of SARS/MERS/COVID-19 infection? Insights from a rapid review of the literature. Int J Environ Res Public Health.

[6] Temsah MH, Al-Sohime F, Alamro N, Al-Eyadhy A, Al-Hasan K, Jamal A, Al-Maglouth I, Aljamaan F, Al Amri M, Barry M, Al-Subaie S (2020). The psychological impact of COVID-19 pandemic on health care workers in a MERS-CoV endemic country. J Infect Public Health.

[7] Saqlain M, Munir MM, Ahmed A, Tahir AH, Kamran S (2020). Is Pakistan prepared to tackle the coronavirus epidemic?. Drugs Ther Perspect.

[8] John Hopkins University. Coronavirus Resource Center. 2020. https://coronavirus.jhu.edu/region/pakistan. Accessed October 1, 2020.

[9] Majeed S, Ashraf M. Psychological impacts of social distancing during COVID-19 pandemic in adolescents of lahore, Pakistan. Ann King Edward Medical University. 2020;26(Special Issue):165–9.

[10] Khalid A, Ali S (2020). COVID-19 and its challenges for the healthcare system in Pakistan. Asian Bioethics Rev.

[11] Ayyaz M, Chima KK, Butt UI, Khan WH, Umar M, Farooka MW, Wasim T (2020). Combating COVID 19 in a public sector hospital in Pakistan. Ann Med Surg.

[12] Raza A, Matloob S, Rahim NF, Halim HA, Khattak A, Ahmed NH (2020). Factors impeding health-care professionals to effectively treat coronavirus disease 2019 patients in Pakistan: a qualitative investigation. Front Psychol..

[13] Liu Y, Ning Z, Chen Y, Guo M, Liu Y, Gali NK, Sun L, Duan Y, Cai J, Westerdahl D, Liu X (2020). Aerodynamic analysis of SARS-CoV-2 in two Wuhan hospitals. Nature.

[14] Chirico F, Magnavita N (2020). Covid-19 infection in Italy: an occupational injury. S Afr Med J.

[15] Yıldırım M, Arslan G, Özaslan A (2020). Perceived risk and mental health problems among healthcare professionals during COVID-19 pandemic: exploring the mediating effects of resilience and coronavirus fear. Int J Ment Health Addict.

[16] Kortum E, Leka S, Cox T (2010). Psychosocial risks and work-related stress in developing countries: health impact, priorities, barriers and solutions. Int J Occup Med Environ Health.

[17] Vévoda J, Vévodová Š, Nakládalová M (2018). Psychosocial risks in healthcare. Casopis lekaru ceskych.

[18] Sethi BA, Sethi A, Ali S, Aamir HS. Impact of Coronavirus disease (COVID-19) pandemic on health professionals. Pak J Med Sci. 2020;36(COVID19-S4):6–11. 10.12669/pjms.36.COVID19-S4.2779 PMC7306959 32582306

[19] Shanafelt T, Ripp J, Trockel M (2020). Understanding and addressing sources of anxiety among health care professionals during the COVID-19 pandemic. JAMA.

[20] Liu Q, Luo D, Haase JE, Guo Q, Wang XQ, Liu S, Xia L, Liu Z, Yang J, Yang BX (2020). The experiences of health-care providers during the COVID-19 crisis in China: a qualitative study. Lancet Glob Health.

[21] Mohsin M, Syed J (2020). The missing doctors – An analysis of educated women and female domesticity in Pakistan. Gender, Work & Organization..

[22] Labrague LJ, DelosSantos JA (2020). COVID-19 anxiety among front-line nurses: predictive role of organisational support, personal resilience and social support. J Nurs Manag..

[23] Sun N, Wei L, Shi S, Jiao D, Song R, Ma L, Wang H, Wang C, Wang Z, You Y, Liu S (2020). A qualitative study on the psychological experience of caregivers of COVID-19 patients. Am J Infect Control.

[24] Mills J, Birks M. Qualitative methodology: A practical guide. Sage; 2014.

[25] Smith JA (1996). Qualitative methodology: analysing participants’ perspectives. Curr Opin Psychiatry.

[26] Farooq MB, De Villiers C (2017). Telephonic qualitative research interviews: when to consider them and how to do them. Meditari Account Res.

[27] Sundler AJ, Lindberg E, Nilsson C, Palmér L (2019). Qualitative thematic analysis based on descriptive phenomenology. Nurs Open.

[28] Chew NW, Lee GK, Tan BY, Jing M, Goh Y, Ngiam NJ (2020). A multinational, multicentre study on the psychological outcomes and associated physical symptoms amongst healthcare workers during COVID-19 outbreak. Brain Behav Immun.

[29] Lissoni B, Del Negro S, Brioschi P, Casella G, Fontana I, Bruni C, Lamiani G (2020). Promoting resilience in the acute phase of the COVID-19 pandemic: psychological interventions for intensive care unit (ICU) clinicians and family members. Psychol Trauma.

[30] Chirico F, Nucera G (2020). An italian experience of spirituality from the coronavirus pandemic. J Relig Health.

[31] Webb A (2015). Quarantine, isolation, and health care workers. Continuum..

[32] Robertson E, Hershenfield K, Grace SL, Stewart DE (2004). The psychosocial effects of being quarantined following exposure to SARS: a qualitative study of Toronto health care workers. Can J Psychiatry.

[33] Sadang JM (2021). The lived experience of Filipino nurses’ work in COVID-19 quarantine facilities: a descriptive phenomenological study. Pacific Rim Int J Nurs Res.

[34] Maunder R, Hunter J, Vincent L, Bennett J, Peladeau N, Leszcz M, Sadavoy J, Verhaeghe LM, Steinberg R, Mazzulli T (2003). The immediate psychological and occupational impact of the 2003 SARS outbreak in a teaching hospital. CMAJ.

[35] Duan L, Zhu G (2020). Psychological interventions for people affected by the COVID-19 epidemic. Lancet Psychiatry.

[36] Fawaz M, Samaha A (2020). The psychosocial effects of being quarantined following exposure to COVID-19: a qualitative study of Lebanese health care workers. Int J Soc Psychiatry.

[37] Robert J, Piemonte N, Truten J (2018). The reflective scribe: Encouraging critical self-reflection and professional development in pre-health education. J Med Humanit.

[38] Raven J, Wurie H, Witter S (2018). Health workers’ experiences of coping with the Ebola epidemic in Sierra Leone’s health system: a qualitative study. BMC Health Serv Res.

[39] Chersich MF, Gray G, Fairlie L, Eichbaum Q, Mayhew S, Allwood B (2020). COVID-19 in Africa: care and protection for frontline healthcare workers. Global Health.

[40] Aacharya RP, Shah A. Ethical dimensions of stigma and discrimination in Nepal during COVID-19 pandemic. Ethics in Med Public Health. 2020;14:100536. 10.1016/j.jemep.2020.100536 PMC7250747 32835054

[41] Ali S, Noreen S, Farooq I, Bugshan A, Vohra F. Risk Assessment of Healthcare Workers at the Frontline against COVID-19. Pak J Med Sci. 2020;36(COVID19-S4):99–103. 10.12669/pjms.36.COVID19-S4.2790 PMC7306961 32582323

