# Supplementary material for: Psychosocial, emotional and professional challenges faced by female healthcare professionals during the COVID-19 outbreak in Lahore, Pakistan: a qualitative study
Source: BMC Womens Health. 2021 May 12;21:197. doi: 10.1186/s12905-021-01344-y (PMC8114660; doi:10.1186/s12905-021-01344-y)
Supplement: Supplementary file 1 — Additional file 1. Interview guide. [file 12905_2021_1344_MOESM1_ESM.pdf]

Date of interview:

Number of interview:

Informed consent:

PSYCHOSOCIAL CHALLENGES FACED BY FEMALE HEALTH CARE PROFESSIONALS DURING COVID-19 OUTBREAK IN LAHORE, PAKISTAN

INTERVIEW GUIDE

Introduction: Self introduction – name and affiliation

Purpose of interview: As COVID-19 pandemic is a new challenge for health care professional all over the world, I am interested in understanding your views and experiences. It covers challenges that you faced as a female HCP while treating COVID-19 patients as well as managing personal and professional life. I am really thankful for your time and willingness for participating in this study. I assure you that your response would be completely anonymous.

Introduction of participant:

How long have you been working as a doctor/nurse/paramedic?

What is your Age, marital status, number of children, family members?

For how much time did you work in COVID -19 dedicated wards/ICU?

What kind of training did you receive for treating COVID-19 patients?

Psychological concerns:

What are your core psychological concerns for treating COVID-19 patients?

What challenges did you encounter and what were your coping methods?

What were the supporting factors from family, community or workplace management during and after your duty in COVID-19 dedicated wards?

Did you or any of your family members got infected during or post COVID-19 duty?

How did you manage your work and family life while handling the pandemic?

What were the societal pressures you faced?

What were your coping strategies?

Self-reflection

As an HCP, how was working with COVID-19 patients, how was it different to usual work?

Has it changed your trust in yourself, your profession, government, or administration?

What are the challenges you faced as a female health care provider at workplace and at home?

After completing your duty, did you got any psychological assessment or therapy for releasing stress?

Do you have any further concerns or recommendations?
